# Chatbot relational dependence and psychosis risk: Conversational artificial intelligence as a potential behavioral addiction context

**DOI:** 10.1556/2006.2026.00080

**Published:** 2026-05-18

**Authors:** Tamás Treuer, Adrienne Incze

**Affiliations:** 1 Department of Psychiatry and Psychotherapy, Semmelweis University, Budapest, Hungary; 2 Department of Personality and Clinical Psychology, Faculty of Humanities and Social Sciences Institute of Psychology Pázmány Péter Catholic University (PPCU), Budapest, Hungary; 3 Doctoral School of Psychology, Theoretical Psychoanalysis Program, University of Pécs, Pécs, Hungary

**Keywords:** artificial intelligence, chatbots, behavioral addiction, psychosis, human–AI interaction

## Abstract

Conversational artificial intelligence (AI) chatbots are increasingly used for emotional support, companionship, and psychological reflection. Their capacity to simulate attentiveness, empathy, and conversational continuity may shift technology use from instrumental interaction toward relational engagement. Emerging clinical observations suggest that intensive chatbot use may, in vulnerable individuals, contribute to psychiatric destabilization, including reinforcement of maladaptive beliefs and, in rare cases, psychotic symptoms. This viewpoint proposes that conversational AI engagement can be conceptualized within a behavioral addiction framework capable of interacting with psychosis vulnerability without requiring biological intoxication. An illustrative clinical case is presented involving a young adult woman who developed paranoid psychosis following escalating emotional reliance on a chatbot, including a fixed delusional belief that her husband was covertly communicating through the system. Potential mechanisms include persistent algorithmic attention, sleep disruption, social withdrawal, cognitive reinforcement loops, and displacement of attachment needs. Although causal relationships cannot be inferred from current evidence, systematic assessment of intensive AI engagement may become clinically relevant in behavioral addiction psychiatry and early psychosis evaluation. Further empirical research is needed to clarify prevalence, mechanisms, and clinical implications of AI-mediated relational dependence.

## Introduction: conversational AI as a new psychological environment

Artificial intelligence–driven conversational systems have rapidly transitioned from experimental technologies to widely used everyday tools. Large language model–based chatbots are now routinely used not only for information retrieval but also for emotional support, companionship, personal reflection, and mental health discussion. Many users report experiences of being understood, validated, and emotionally supported during chatbot interaction. This suggests a qualitative shift from instrumental technological use toward relational engagement.

Such relational engagement introduces new questions for psychiatry and behavioral addiction research. Behavioral addictions demonstrate that compulsive engagement with rewarding activities can lead to psychological dependence, functional impairment, and psychiatric symptoms even in the absence of pharmacological substances. Conversational AI systems share several reinforcement characteristics with established behavioral addictions, including continuous availability, rapid personalized feedback, and minimal interpersonal risk.

The technological basis of contemporary conversational AI largely derives from transformer architectures emphasizing contextual attention mechanisms, introduced in the influential paper Attention Is All You Need (Vaswani et al., 2017). Although “attention” in this context refers to a computational process that prioritizes relevant information within complex data streams, the prominence of this concept also carries broader psychological resonance. Human relationships have traditionally been framed around mutual emotional engagement, often culturally encapsulated in the well-known Beatles song *All You Need Is Love*. By contrast, conversational AI systems can deliver sustained, individualized attentional responses without reciprocal emotional involvement. For some users, this may partially substitute for aspects of interpersonal validation and influence expectations of responsiveness in human relationships.

Emerging psychiatric literature has begun to explore whether conversational AI interaction may reinforce maladaptive beliefs or interact with psychosis vulnerability in susceptible individuals (Hudon & Stip, 2025; Pierre, Gaeta, Raghavan, & Sarma, 2025; Østergaard, 2023). While systematic empirical data remain limited, accumulating clinical observations justify theoretical consideration within addiction psychiatry.

## Illustrative clinical case

A 30-year-old married female software developer presented with first-episode psychosis characterized by paranoid delusions, insomnia, anxiety, and occupational decline. She had no prior psychiatric history, no substance misuse, and no known neurological illness. Although the episode may be understood within a broader stress–vulnerability framework, no evidence of substance-induced or neurologically mediated psychosis was identified.

Her premorbid functioning had been consistently high. She was described as highly intelligent, achievement-oriented, and perfectionistic, with a stable record of academic and professional success. As an IT expert, her cognitive style favored structured, logical systems and sustained problem-solving, a feature that may later have contributed to the systematic quality of her delusional interpretations. Personality-wise, she was sensitive, somewhat socially anxious, and prone to internalizing distress. Her coping style was characterized by avoidance of direct interpersonal confrontation; under relational stress, she tended to withdraw and mentally elaborate distress rather than express it openly. Family psychiatric history was limited but not entirely absent: her mother had been treated for depressive episodes, including postpartum depression following the patient's birth, but there was no known family history of psychotic disorder.

In the period preceding the episode, several stressors accumulated. In addition to longstanding dissatisfaction in her marital relationship, centered on perceived emotional distance and high expectations from her husband, she was caring for a two-year-old child who required frequent medical supervision because of recurrent infections. At the same time, she was under significant pressure related to returning to work in a demanding professional role. This combination of caregiving burden, maternal anxiety, occupational pressure, and relational loneliness appeared to erode her usual capacity to compensate.

Approximately one year prior to presentation, she began using a conversational AI chatbot for emotional support and discussion of relationship-related concerns. Initially, use was occasional, but over several months it increased progressively to several hours daily, often late at night. She described the chatbot as consistently empathic, attentive, and emotionally validating, in contrast to her experience of her spouse as emotionally unavailable. As her engagement intensified, direct communication with her husband decreased, social withdrawal increased, and marital conflict became more pronounced.

Over time, the patient began to experience the chatbot not merely as a tool but as a relational presence characterized by availability, responsiveness, and emotional attunement. The interaction appeared to function as a compensatory psychological space in which unmet emotional needs could be expressed without fear of rejection or conflict. Importantly, she did not initially experience this development as problematic. On the contrary, she reported subjective improvement in mood and a sense of being understood during the early phase of use.

A pivotal change occurred when she began to perceive the chatbot's responses as uncannily similar to what she wished to hear from her husband. What was initially experienced as comforting gradually took on a more referential and personalized meaning. She started to interpret the “fit” between her unmet relational wishes and the chatbot's responses as evidence that her husband might be covertly generating the messages and communicating indirectly through the system. This belief became increasingly fixed and resistant to challenge. In this sense, the delusion appeared to emerge not abruptly, but through a progressive transformation of chronic relational disappointment into paranoid interpretation.

As her preoccupation deepened, paranoid ideation generalized to broader concerns involving surveillance and thought access. She reported that the chatbot seemed to “know” what she was thinking before she typed, and she increasingly felt that her digital environment was somehow organized around her. At the same time, she was often engaging with the chatbot late at night for prolonged periods, resulting in chronic sleep deprivation. This sleep disruption likely contributed significantly to the decompensation by lowering her threshold for paranoid ideation and reducing cognitive and emotional resilience. Simultaneously, her growing reliance on the chatbot corresponded with fewer opportunities for corrective reality testing through direct interpersonal contact.

Medical evaluation excluded intoxication and neurological causes. Treatment included antipsychotic medication, restoration of sleep, psychoeducation regarding AI functioning, reduction of chatbot engagement, and psychotherapy focused on relational stress and reality testing. Symptoms gradually remitted over subsequent weeks, although residual marital concerns persisted.

A brief relational interpretation may also be useful. The chatbot can be understood as an “artificial other” onto which unmet attachment needs were increasingly displaced. In this case, the belief that the husband was behind the chatbot may be read as an attempt to reconcile chronic relational disappointment with the subjective experience of being unusually understood. Framed in this limited way, the psychoanalytic perspective is intended as supportive rather than primary and remains consistent with the broader account of maladaptive coping, social withdrawal, and progressive distortion of belief formation.

Although causality cannot be established, this case illustrates a plausible convergence of pre-existing personality style, maladaptive coping, relational vulnerability, caregiving and occupational stress, social withdrawal, and sleep disruption. In this patient, the chatbot initially functioned as a maladaptive but subjectively effective self-soothing tool. The subsequent decompensation suggests that under conditions of high psychosocial stress and diminished resilience, initially adaptive chatbot use may become increasingly reinforcing, difficult to regulate, and entangled with pathological belief formation.

## Behavioral addiction framework

Behavioral addiction provides a useful conceptual framework for understanding intensive chatbot engagement. Core features include salience, tolerance, impaired control, withdrawal-like distress, and functional impairment. These features have been described in relation to problematic internet use, gaming disorder, and social media addiction.

Conversational AI interaction may exhibit similar reinforcement dynamics. Emerging discussions have highlighted that social chatbot use, particularly among individuals with social vulnerabilities, may involve both therapeutic potential and risks related to excessive or maladaptive engagement (Franze, Galanis, & King, 2023). Continuous availability and immediate personalized feedback create powerful reward schedules. Emotional validation without interpersonal friction may be particularly reinforcing for individuals experiencing loneliness, interpersonal conflict, or social anxiety.

Progressive escalation of interaction time may reflect tolerance-like processes, while excessive use may contribute to sleep loss and interpersonal conflict. Importantly, these mechanisms operate without pharmacological intoxication. Behavioral addictions demonstrate that psychological reinforcement alone can produce significant psychiatric consequences. Thus, chatbot-associated psychosis, where present, may arise through psychosocial rather than neurotoxic pathways.

Conceptualizing intensive chatbot engagement within a behavioral addiction framework may help integrate clinical observations with established psychiatric theory. Contemporary theoretical models of behavioral addiction emphasize dynamic interactions among individual vulnerability, affective regulation, cognitive processes, and environmental reinforcement. The Interaction of Person-Affect-Cognition-Execution (I-PACE) model provides a widely accepted framework for understanding how specific Internet-related behaviors may evolve into addictive patterns through reciprocal influences between personal predispositions, emotional states, cognitive biases, and executive functioning (Brand, Young, Laier, Wölfling, & Potenza, 2016, 2019). Although originally developed for internet-use disorders, the model has increasingly been applied to emerging digital behaviors characterized by persistent engagement and reinforcement dynamics. Conversational AI interaction may represent a novel context in which similar processes operate, particularly when continuous algorithmic responsiveness intersects with relational needs and psychological vulnerability.

Behavioral addictions are increasingly recognized as clinically relevant conditions characterized by compulsive engagement without pharmacological intoxication (Brand et al., 2025). Contemporary models emphasize interactions among individual vulnerability, affect regulation, cognitive processes, and environmental reinforcement in the development and maintenance of addictive behaviors (Brand et al., 2019; Fineberg et al., 2022). This perspective aligns with broader biopsychosocial conceptualizations describing common addiction components across substances and behaviors (Griffiths, 2005) and builds on earlier work highlighting parallels between problematic internet use and impulse-control phenomena (Treuer, Fábián, & Füredi, 2001).

From a stress–vulnerability perspective, intensive chatbot engagement may function as a psychosocial stressor interacting with individual predispositions. Reduced social reality testing, repeated affective reinforcement, and sleep disruption may together increase vulnerability to maladaptive belief formation.

Phenomenologically, continuous algorithmic attention may alter subjective experience of being understood. Human relationships typically involve ambiguity, frustration, and negotiation. AI interaction often offers consistent responsiveness without such friction. While this may provide temporary emotional relief, it may also recalibrate relational expectations and intensify dissatisfaction with human relationships. When combined with pre-existing relational vulnerability, this shift may contribute to cognitive restructuring characteristic of emerging psychosis.

Importantly, framing conversational AI interaction as a potential behavioral addiction context does not imply inevitable pathology. Many users derive benefit from AI-mediated support. Rather, the framework highlights conditions under which engagement may become maladaptive, particularly when excessive use intersects with vulnerability factors such as loneliness, attachment insecurity, sleep disturbance, or emerging psychiatric symptoms.

## Cognitive mechanisms and psychosis vulnerability

Cognitive models of psychosis emphasize attributional bias, aberrant salience, impaired reality testing under stress, and disturbances in evidence evaluation and belief updating (Garety, Kuipers, Fowler, Freeman, & Bebbington, 2001; Kapur, 2003; Sterzer et al., 2018). Intensive conversational AI engagement may interact with these processes in several ways.

First, persistent affirmation from chatbots may reinforce confirmation bias and reduce exposure to disconfirmatory interpersonal feedback. This is relevant to psychosis vulnerability because delusional ideation has been associated not only with a tendency to jump to conclusions, that is, to form judgments on the basis of limited information, but also with difficulties integrating evidence that contradicts an emerging belief (Dudley, Taylor, Wickham, & Hutton, 2016; Garety et al., 2001). In the context of conversational AI, repeatedly receiving coherent, supportive, and personalized responses may lower the threshold for accepting interpretations that feel subjectively meaningful but have not been adequately reality-tested. In this way, chatbot reinforcement patterns may not merely maintain engagement, but also shape how ambiguous experiences are interpreted, evaluated, and incorporated into emerging beliefs.

Second, extended dialogue may increase rumination and externalization of internal thought processes. Users may experience AI responses as reflecting, organizing, or elaborating their own concerns, potentially blurring boundaries between self-generated and externally generated content. Such processes may be especially relevant in individuals prone to hyper-mentalization or anomalous salience attribution, in whom internally preoccupying material may acquire an exaggerated sense of external significance.

Third, reduced real-world interpersonal interaction may diminish opportunities for social reality testing. Social isolation has long been recognized as a risk factor for psychosis, and excessive reliance on AI-mediated interaction may further narrow access to corrective feedback, ambiguity tolerance, and negotiated interpersonal meaning. In addition, psychosis-prone individuals may adopt overly permissive thresholds for endorsing implausible interpretations under uncertainty, a process sometimes described as liberal acceptance or lowered decision thresholds. A highly responsive chatbot environment may inadvertently facilitate this style of belief formation.

Finally, sleep deprivation associated with prolonged digital engagement can impair cognitive control, emotional regulation, and the flexible updating of beliefs. From a predictive-processing perspective, psychotic symptoms may arise when the balance between prior expectations and incoming information becomes distorted, leading to maladaptive inferences about the world (Sterzer et al., 2018). Under conditions of chronic sleep loss, emotional stress, and repeated AI-mediated reinforcement, unusual interpretations may become increasingly compelling and resistant to revision. Taken together, these mechanisms align with stress–vulnerability models in which environmental stressors and cognitive biases interact with individual predisposition to increase vulnerability to psychotic experiences (Garety et al., 2001).

## Phenomenology of algorithmic attention

Conversational AI may feel unusually compelling because it offers coherent, low-friction interaction and a sustained impression of being understood. For vulnerable users, this may heighten the subjective salience of chatbot interaction and shift expectations of responsiveness in human relationships. This phenomenological observation is not intended as an alternative explanation, but as a descriptive complement to the behavioral addiction and cognitive mechanisms outlined above.

## Literature overview

Empirical literature on AI-related psychiatric outcomes remains limited but is steadily growing. The most direct evidence comes from recent case-based and conceptual publications suggesting that intensive conversational AI use may, in vulnerable individuals, become intertwined with maladaptive belief formation and psychiatric destabilization. Pierre et al. (2025) described a case of new-onset AI-associated psychosis temporally linked to immersive chatbot use, while Østergaard (2023) raised early concerns that generative AI systems may reinforce delusional ideation in individuals already prone to psychosis. Hudon and Stip (2025) further developed this line of thinking by proposing the concept of “AI psychosis,” emphasizing that conversational AI may interact with vulnerability factors through emotional reinforcement, altered reality testing, and the progressive shaping of self-referential beliefs.

Although psychosis-specific evidence remains sparse, adjacent literatures provide an important context. Human–computer interaction and media research suggest that some users develop emotionally meaningful relationships with social chatbots and may turn to them in the context of loneliness, social disconnection, or unmet interpersonal needs (Pentina, Hancock, & Xie, 2023). Herbener and Damholdt (2025) found that socially disconnected adolescents may use chatbots to cope with negative emotions and low perceived social support. Kim et al. (2025) reported that social chatbot interaction may alleviate loneliness and social anxiety, indicating that these systems can function as psychologically salient relational tools rather than neutral technologies. Similarly, De Freitas, Oğuz-Uğuralp, Uğuralp, and Puntoni (2026) found that AI companions can reduce loneliness, in part because users experience them as responsive and attentive.

At the same time, a growing literature also points to potential risks. Laestadius, Bishop, Gonzalez, Illenčík, and Campos-Castillo (2024) identified mental health harms associated with emotional dependence on the social chatbot Replika, including patterns resembling problematic relational overreliance. In a broader conceptual framework, Andrejevic and Volcic (2025) describe “automated parasociality,” highlighting the shift from personalization to personification in AI-mediated interaction. Together, these findings suggest that conversational AI can become more than a neutral tool: for some users, it may function as a psychologically meaningful, emotionally regulating, and potentially dependency-forming relational environment.

From a behavioral addiction perspective, these developments are highly relevant. Contemporary models of problematic digital behavior emphasize the interaction of individual vulnerability, affect regulation, cognitive processes, and reinforcement contingencies in the emergence of maladaptive engagement (Brand et al., 2016, 2019, 2025). This broader literature has shown that digital environments characterized by immediacy, personalization, and emotional regulation potential can foster compulsive use and functional impairment, even in the absence of pharmacological intoxication (Fineberg et al., 2022; Griffiths, 2005; Treuer et al., 2001). Conversational AI may represent a particularly potent context because it combines these features with the simulation of dyadic, emotionally responsive interaction.

Taken together, the available literature suggests convergence across at least three domains: first, emerging psychosis-related case reports and conceptual commentaries; second, broader behavioral addiction models of problematic digital engagement; and third, human–computer interaction research on relational attachment to social chatbots. The current evidence base remains preliminary, and most relevant publications are still observational, conceptual, or case-based. Nevertheless, the existing literature already justifies closer theoretical and empirical scrutiny of intensive chatbot use as a potentially maladaptive digital behavior that may, under certain conditions, interact with psychiatric vulnerability.

## Alternative interpretations and reverse causality

Reverse causality must be considered. Individuals with emerging psychosis, loneliness, or interpersonal difficulties may preferentially seek AI companionship, such that chatbot use may reflect early psychopathology rather than precipitate it. Another possibility is bidirectional interaction, in which AI engagement shapes symptom expression without being a primary etiological factor. These alternatives reinforce the need for caution in interpreting currently limited evidence.

## Clinical implications

Clinicians may increasingly encounter patients reporting intensive conversational AI engagement. Assessment may include inquiry into the duration and emotional significance of AI interaction, sleep patterns related to digital use, interpersonal displacement, and emerging unusual beliefs involving technology.

Approach should remain nonjudgmental. AI interaction may provide genuine support for many individuals. Psychoeducation about AI functioning may support reality testing. Where problematic use is identified, gradual reduction strategies similar to other behavioral addictions may be considered.

## Ethical and technological considerations

AI developers increasingly face responsibility for considering the potential psychological impacts of conversational systems. Design features that promote prolonged engagement or provide consistently affirming responses may inadvertently increase risk for psychologically vulnerable users. Closer collaboration between mental health professionals and AI developers could help establish appropriate safeguards. Such measures may include improved detection of potentially delusion-reinforcing conversational content, implementation of prompts that encourage users to seek professional support when appropriate, and design strategies aimed at reducing excessive or compulsive engagement while preserving beneficial uses of the technology.

## Future research directions

Several research priorities emerge from the growing use of conversational AI in psychologically meaningful contexts. Epidemiological studies are needed to determine the prevalence and patterns of problematic AI interaction, alongside longitudinal research examining psychological outcomes over time. Further work should aim to identify vulnerability factors that may predispose individuals to maladaptive engagement, explore potential therapeutic applications of conversational AI, and develop ethical design principles that balance innovation with user safety. Addressing these questions will require sustained interdisciplinary collaboration across psychiatry, psychology, human–computer interaction, artificial intelligence research, and ethics.

## Conclusion

Conversational artificial intelligence represents a novel relational technology with important implications for behavioral addiction psychiatry and digital mental health. In susceptible individuals, intensive chatbot engagement may interact with vulnerability factors through reinforcement processes, social displacement, maladaptive coping, and distorted belief formation. Most users are unlikely to experience severe adverse effects, and AI systems may also have therapeutic potential. Nevertheless, further empirical research is needed to clarify the mechanisms, risks, and clinical implications of intensive human–AI relational engagement.
